# Evaluation of the Xpert® MTB/RIF assay and microscopy for the diagnosis of *Mycobacterium tuberculosis* in Namibia

**DOI:** 10.1186/s40249-016-0213-y

**Published:** 2017-01-11

**Authors:** Rooyen T. Mavenyengwa, Emma Shaduka, Innocent Maposa

**Affiliations:** 1 Department of Health Sciences, School of Health and Applied Sciences, Namibia University of Science and Technology, Private Bag 13388, Windhoek, Namibia; 2 Department of Medical Microbiology, University of Zimbabwe College of Health Sciences, P. O. Box A178, Harare, Zimbabwe; 3 Department of Mathematics and Statistics, Namibia University of Science and Technology, Private Bag 13388, Windhoek, Namibia

**Keywords:** Tuberculosis, Xpert® MTB/RIF assay, Microscopy, Diagnostic tests, Rifampicin, Namibia

## Abstract

**Background:**

Tuberculosis (TB) kills approximately two million people and infects around nine million worldwide annually. Its proper management, especially in resource-limited settings, has been hindered by the lack of rapid and easy-to-use diagnostic tests. Sputum smear microscopy remains the cheapest, readily available diagnostic method but it only identifies less than half of the patients with a HIV/TB co-infection because the bacilli would have disseminated from the lungs to other areas of the body. The fully automated Xpert® MTB/RIF assay is a promising innovation for diagnosing TB and detecting resistance to rifampicin. This study aimed to evaluate the use of Xpert® MTB/RIF assay and microscopy in the diagnosis of *Mycobacterium tuberculosis* in Namibia, by determining the disease’s epidemiology and calculating the proportion of cases infected just with TB and those with a resistance to rifampicin among the total suspected cases of TB in the country.

**Methods:**

This retrospective study analysed TB cases that were diagnosed using both the Xpert® MTB/RIF assay and microscopy. Data were collected from patient records from the Meditech laboratory information system of the Namibia Institute of Pathology for the time period of July 2012–April 2013. Data from 13 regions were collected.

**Results:**

The total number of specimens collected from patients with symptoms of pulmonary TB was 1 842. Of these, 594 (32.20%) were found to be positive for MTB by Xpert® MTB/RIF assay, out of which 443 (24.05%) were also found to be positive by microscopy. The remainder was negative. The male patients were more resistant to rifampicin when compared to the female patients.

**Conclusions:**

Tuberculosis is widely distributed throughout Namibia, with slightly more males infected than females. Most TB patients are also co-infected with HIV. Both microscopy and Xpert® MTB/RIF assay are crucial for the diagnosis of TB in the country. Screening diagnostic efforts should focus on the sexually active HIV positive male population who could be the source of more RIF-resistant TB than females to prevent its spread.

**Electronic supplementary material:**

The online version of this article (doi:10.1186/s40249-016-0213-y) contains supplementary material, which is available to authorized users.

## Multilingual abstracts

Please see Additional file 1 for translations of the abstract into the five official working languages of the United Nations

## Background

Tuberculosis (TB), an infection that mainly affects the lungs, is caused by *Mycobacterium tuberculosis* (MTB). The infection can be diagnosed by sputum smear microscopy, chest radiography, culture-based testing, or nucleic acid amplification tests (NAATs) [1, 2]. Recently, new diagnostic techniques have been discovered, including interferon-gamma release assays and Xpert® MTB/RIF assay [3].

The elimination of TB requires improved detection and early treatment. Many people still die from TB due to delayed diagnosis and this contributes to the risk of TB transmission [4]. Microscopy with Ziehl-Neelsen (ZN) staining is the most common and often the only laboratory technique used to diagnose TB in most developing countries [1, 5–8]. However, errors associated with microscopy lead to misdiagnoses [6]. It is therefore vital to improve and possibly replace microscopy with simpler, more affordable, and more accurate diagnostic methods. Patients who are co-infected with HIV are often the ones whose smear results are negative, when in fact they actually have TB [9]. This is because the bacilli would have disseminated from the lungs to other organs due to immunosuppression. If facilities are available, it would be best to use the gold standard of diagnosing pulmonary TB using culture-based testing in HIV positive patients [10].

GeneXpert is a system based on a NAAT that rapidly diagnoses and detects MTB and rifampicin (RIF) resistance in clinical specimens [4, 9, 11]. The World Health Organization endorsed it as the initial diagnostic test for individuals suspected of having multidrug-resistant TB (MDR-TB) or HIV-associated TB [7, 12, 13]. However, Xpert® MTB/RIF assay cannot completely eliminate the need for microscopy and culture, as the latter are essential for the detection of resistance to drugs other than RIF [14].

Namibia is the country with the fourth highest TB burden in the world. The incidence rate in 2015 was 651/100 000 [15]. Cases of TB in Namibia are widely distributed throughout the country, however, the majority of cases are reported in the regions of Khomas, Ohangwena, Erongo, and Kavango [15, 16]. Currently, the public health reference diagnostic laboratory, the Namibia Institute of Pathology (NIP), uses ZN microscopy, Xpert® MTB/RIF assay, and, to a lesser extent, TB culture to diagnose TB.

The aim of the study was to evaluate the use of ZN microscopy and Xpert® MTB/RIF assay for the diagnosis of TB cases in Namibia. This was done by determining the disease’s epidemiology and calculating the proportion of TB positive cases and cases with resistance to RIF among the sputum samples stored at the NIP in Windhoek.

## Methods

### Study design and setting

This was a retrospective study that analyzed MTB cases diagnosed using both the Xpert® MTB/RIF assay (Cepheid, Sunnyvale, CA, USA) and microscopy. Data were collected from patient records that were retrieved from the Meditech laboratory information system of the NIP for the period of July 2012–April 2013. The records came from all 13 regions of Namibia, but excluded data from private hospitals and clinics. The specimens were tested for acid-fast bacilli using microscopy and Xpert® MTB/RIF assay within three days of being received by the laboratory. The total number of specimens collected from patients with symptoms of pulmonary TB was 1 842.

### Data collection

The data extracted included: HIV status based on the Determine™ HIV-1/2 test (Alere, Milan, Italy), laboratory number, age, sex, positive and negative results from microscopy and Xpert® MTB/RIF assay testing, and the patient’s region as indicated by the given residential address. Data were entered and sorted using Microsoft Excel before being analyzed using IBM SPSS version 21.0 (SPSS Inc., Chicago, Illinois, USA). If the p-value was less than 0.05, this was considered as significant.

## Findings

Cases were categorized as either sputum smear-positive or sputum smear-negative. They were also categorized as either Xpert® MTB/RIF assay positive or Xpert® MTB/RIF assay negative, based on the assay results. Out of the 1 842 specimens tested, 594 (32.20%) were found to be positive for MTB by Xpert® MTB/RIF assay, and of those 443 (24.05%) were also found to be positive by microscopy. The remainder was negative.

Table 1 summarizes the numbers of MTB cases detected by microscopy in the 13 regions of Namibia with regard to sex. Khomas had the highest number of MTB cases, followed by Oshana and Ohangwena. The lowest numbers of cases were recorded in Zambezi and Omaheke. The specific regions where fifteen women resided were not indicated in the data set and hence were missing. We also compared the results from the smear microscopy by sex. Microscopy detected that there were slightly more males with MTB than females. The *P*-value obtained using the chi-square test was 0.489, indicating that there was no significant association between smear microscopy and sex.

**Table 1:** Positive MTB cases detected by microscopy in Namibia from July 2012 to April 2013, by region and sex

Region	Sex				Total	
	Male		Female			
	No.	%	No.	%	No	%
Khomas	28	14.7	47	18.9	75	17.1
Kavango	19	10.0	12	4.8	31	7.1
Oshikoto	32	16.8	22	8.8	54	12.3
Ohangwena	25	13.2	34	13.7	59	13.4
Kunene	2	1.1	4	1.6	6	1.4
llKaras	10	5.3	10	5.3	31	7.1
Oshana	25	13.2	48	19.3	73	16.6
Otjozondjupa	5	2.6	11	4.4	16	3.6
Erongo	11	5.8	11	4.4	22	5
Hardap	4	2.1	10	4	14	3.2
Omusati	25	13.2	27	10.8	52	11.1
Zambezi	2	1.1	0	0	2	0.5
Omaheke	2	1.1	2	0.8	4	0.9
Total	190	100	238	100	428	100

The majority of the patients belonged to the age groups of 20–40 years and 41–60 years. In the age group of above 60 years, there was a lower number of MTB cases, but the lowest number of MTB cases was recorded among the age group of below 20 years (see Table 2). Data on the age of four patients were not available because it was missing from the data set. Of the total 249 MTB-positive males, 190 (76.3%) tested positive for MTB using both methods and the rest tested positive by Xpert® MTB/RIF assay only. Out of the females, 238 (95.2%) tested positive for MTB using both methods, with an extra 12 testing positive using Xpert® MTB/RIF assay only.

**Table 2:** Comparison of sputum smear-positive results and positive Xpert® MTB/RIF assay results, by age

Age group		Xpert® result	
		Positive	
		No.	%
Below 20 years	Sputum smear-positive	10	62.5
	Total	16	100.0
Between 20 and 40 years	Sputum smear-positive	210	78.9
	Total	266	100.0
Between 40 and 60 years	Sputum smear-positive	169	71.3
	Total	237	100.0
Above 60 years	Sputum smear-positive	51	71.8
	Total	71	100.0
Total	(defined as Xpert® positive)	440	74.6
	Total	590	100.0

Of the total positive MTB cases, 341 (57.4%) patients also tested positive for HIV and 137 (23.1%) tested negative for HIV. The HIV status of the remaining 116 (19.5%) patients was unknown (see Table 3). Patients in the age groups of 20–40 years and 41–60 years were found to be the most co-infected with MTB/HIV (45.7% and 49.0% HIV, respectively). Only 18 (5.3%) of the total HIV-positive patients belonged to the age group of above 60 years. There were no HIV-positive patients among the age group of below 20 years.

**Table 3:** Comparison of sputum smear-positive and Xpert® MTB/RIF assay results, by HIV status

HIV Status		Within Xpert® result	
		Positive	
		No.	%
Positive	Sputum smear-positive	263	77.1
	Total	341	100.0
Negative	Sputum smear-positive	102	74.5
	Total	137	100.0
Unknown	Sputum smear-positive	78	67.2
	Total	116	100.0
Total	(defined as Xpert® positive)	443	74.6
	Total	594	100.0

Table 4 summarizes the distribution of HIV in MTB-infected patients, by sex. A higher percentage of HIV-positive males (63.9%) as compared to females (52.8%) was recorded.

**Table 4:** HIV co-infection in MTB-infected patients diagnosed using the Xpert® MTB/RIF assay, by sex

		HIV STATUS			Total
		Positive	Negative	Unknown	
Sex					
Male	No.	159	45	45	249
	%	63.9	18.1	18.1	100.0
Female	No.	182	92	71	345
	%	52.8	26.7	20.5	100.0
Total	No.	341	137	116	594
	%	57.4	23.1	19.5	100.0

The number of MTB cases and RIF-resistant cases diagnosed using the Xpert® MTB/RIF assay with regard to sex is presented in Table 5. From the total of 594 MTB-positive patients, 27.9% were resistant to RIF and 72.1% were sensitive to it. More males were found to be resistant to RIF as compared to the females, with the difference statistically significant (*P* = 0.023).

**Table 5:** Summary of MTB cases and RIF-resistant cases diagnosed using the Xpert® MTB/RIF assay, by sex

		Rifampicin		Total
		Resistant	Sensitive	
Sex				
Male	No.	86	163	249
	%	34.5	65.5	100
Female	No.	80	265	345
	%	23.8	76.2	100
Total	No.	166	428	594
	%	27.9	72.1	100

## Discussion

A total of 594 specimens tested positive for MTB using the Xpert® MTB/RIF assay, and of these 443 (74.6%) also tested positive using smear microscopy. These data indicate that smear microscopy detected TB in fewer patients than Xpert® MTB/RIF assay. Smear microscopy might miss specimens with low bacilli/ml, as direct smear microscopy needs at least 5 000 bacilli/ml of sputum for direct microscopy to show a positive result [1, 11].

In the current study, there were slightly more females with stronger smear-positivity than males and the difference was not statistically significant. Although culture-based testing is the gold standard for TB diagnosis, it needs high-safety settings and facilities that are expensive to maintain, and this means that most low- and middle- income countries cannot afford to do this kind of testing. For this reason, sputum smear microscopy remains the main and often only diagnostic tool for MTB [6–8, 17, 18]. Tuberculosis culture for drug-susceptibility testing is not routinely performed in Namibia [19]. Culture-based testing is also quite time-consuming, requiring more than eight weeks for results to become available. However, the Xpert® MTB/RIF assay gives accurate results in a shorter time period of less than 2 h and being cheaper than culture is therefore more appropriate for use in low- and middle-income countries [20]. That being said, it cannot completely replace culture-based testing, as it only detects RIF resistance and does not provide drug resistance profiles for other TB drugs. Namibia needs to scale up its facilities for culture-based testing so that specimens that are found to be RIF-resistant are further tested for MDR-TB and extensively drug-resistant (XDR) TB. This will assist in guiding treatment regimens for patients who might have MDR-TB or XDR TB.

This study found that MTB infection is distributed throughout Namibia, with males slightly more infected than females. Khomas was the region with the highest number of MTB cases, whereas Zambezi and Omaheke had the lowest numbers of MTB cases (each). This is similar to findings from a previous study, which also showed that TB was distributed all over Namibia with the highest number of cases reported in Khomas and Okavango [16]. Khomas had the highest number of MTB cases possibly because of its central location, which includes the densely populated capital city Windhoek, where there is also a high HIV prevalence. HIV predisposes patients to TB. This region is receiving information on MTB testing awareness from different health-related stakeholders, which likely resulted in more people with TB getting tested once they suspected that they had TB symptoms. Zambezi and Omaheke recorded fewer MTB-positive patients probably due to their remote locations. Those who could be TB positive rarely have the information that could make them question or suspect that they are TB positive.

This study found that the age group with the highest MTB infection were those aged between 20 and 40 years (44.8%). The age group of below 20 years had the least amount of people with MTB infection (2.7%). The low infection rate in the younger age group could possibly be explained by this age group’s literacy, which allows them to effectively receive educational information on MTB prevention from schools and the media.

The study found a high MTB/HIV co-infection rate of 57.4% emphasizing the need for proactive and rapid screening of MTB/HIV co-infected patients. The number of patients with MTB/HIV in the age groups of 20–40 years was found to be high because the age group is considered a sexually active age group, with members having an increased risk of contracting HIV through unsafe sex [21]. Most of the HIV-positive patients in our study belonged to that age group.

Xpert® MTB/RIF assay is an important initial diagnostic test for patients suspected of having MDR-TB and those with a MTB/HIV co-infection [18]. An association between sex and sensitivity to RIF was noted. A smaller number of females was infected with a RIF-resistant strain than males possibly because females better adhere to drug taking than males. Fewer females than males are known to take alcohol or drugs in Namibia which often contributes to poor drug adherence. However, there could also be other reasons for this such as TB transmission mechanisms, primary versus secondary drug resistance, different co-infections, and different health-seeking behaviors. For instance men tend to socialize in crowded places than females such that they are likely to get community-acquired resistant strains. Further investigations are therefore needed. In 2014, only 137 cases of MDR-TB have been reported, indicating that drug susceptibility testing is improving in Namibia [15].

## Conclusions

Tuberculosis is widely distributed throughout Namibia, with more males infected than females. Males have more resistance to RIF than females. Tuberculosis infection was mainly common in the age group of 20–40 years. A large proportion of MTB patients were also infected with HIV. Microscopy and Xpert® MTB/RIF assay are both crucial MTB diagnostic tools in areas of high HIV incidence. Screening diagnostic efforts should focus on the sexually active male HIV positive population who could be the source of more RIF-resistant TB than females to prevent its spread.

## Additional file

Additional file 1: Multilingual abstracts in the five official working languages of the United Nations. (PDF 783 kb)

## Abbreviations

MDR-TB: Multidrug-resistant TB
MTB: *Mycobacterium tuberculosis*
NAAT: Nucleic acid amplification test
NIP: Namibia Institute of Pathology
RIF: Rifampicin
TB: Tuberculosis
XDR TB: Extensively drug-resistant TB
ZN: Ziehl-Neelsen
