# Supplementary material for: Evaluation of the Xpert® MTB/RIF assay and microscopy for the diagnosis of Mycobacterium tuberculosis in Namibia
Source: Infect Dis Poverty. 2017 Jan 11;6:13. doi: 10.1186/s40249-016-0213-y (PMC5237317; doi:10.1186/s40249-016-0213-y)
Supplement: Additional file 1: — Multilingual abstracts in the five official working languages of the United Nations. (PDF 783 kb) [file 40249_2016_213_MOESM1_ESM.pdf]

Translation of the abstract into the five official working languages of the United Nations

تقييم تحليل واختبار تأثير والفحص المجهري لعقار **Xpert® MTB/RIF** لتشخيص المتفطرة السُلية في ناميبيا

روبين ت. مافنينجوا، إما شادوكا، وإنوسنت مابوسا

Rooyen T. Mavenyengwa, Emma Shaduka, Innocent Maposa

ملخص

الخلفية: يتسبب السُل في وفاة حوالي اثنين مليون شخصًا ويصيب حوالي تسعة ملايين شخصًا سنويًا على مستوي العالم. وقد تسبب غياب الاختبارات التشخيصية السريعة وسهلة الاستخدام في إعاقة احتواء المرض ولا سيما في الأماكن ذات الموارد المحدودة. ويبقى تحليل مزرعة البصاق الأداة التشخيصية الأرخص والأكثر إتاحة إلا أنها تقوم بتشخيص أقل من نصف المرضى المصابين بعدوى مرافقة لمرضي نقص المناعة والسُل لأن بكتيريا العُصيات ستكون قد انتشرت من الرئتين إلى مناطق أخرى من الجسم. إن تحليل **Xpert® MTB/RIF** لهو ابتكار واعد لتشخيص الإصابة بالسُل واكتشاف المقاومة للريفامبيسين. وتهدف هذه الدراسة إلى تقييم استخدام التحليل والفحص المجهري لـ **Xpert® MTB/RIF** لتشخيص المُتفطرة السُلية في ناميبيا، من خلال تحديد علم الوبائيات الخاص بالمرض واحتساب نسبة الحالات المُصابة فقط بمرض السُل والحالات المقاومة للريفامبيسين بين إجمالي الحالات المُشتبه في إصابتها بمرض السُل في البلاد.

الوسائل: تحلل تلك الدراسة الاستعادية حالات الإصابة بالسُل التي تم تشخيصها باستخدام كلٍ من التحليل والفحص المجهري لـ **Xpert® MTB/RIF**. تم جمع البيانات من سجلات المرضى الموجودة بنظام المعلومات لمعمل Meditech بالمعهد النامیبي لعلم الأمراض عن الفترة الزمنية من يوليو ٢٠١٢ إلى ابريل ٢٠١٣. تم جمع البيانات من ١٣ منطقة.

النتائج: بلغ اجمالي عدد العينات التي تم جمعها من مرضى ظهرت عليهم أعراض مرض السُل الرئوي ١٨٤٢ عينة، منهم ٥٩٤ حالة (٣٢,٢٪) أثبت تحليل **Xpert® MTB/RIF** أنها إيجابية وحاملة للمتفطرة السُلية، منهم ٤٤٣ حالة (٢٤,٠٥٪) تم اثبات حملهم للمرض عن طريق الفحص المجهري. أما بقية الحالات فكانت سلبية. وقد كان المرضى الذكور أكثر مقاومة للريفامبيسين مقارنة بالإناث.

الاستنتاجات: إن مرض السُل منتشر انتشارًا واسعًا في كافة أنحاء ناميبيا، ويزيد عدد المصابين الذكور قليلًا عن الإناث. كما أن معظم مرضى السّل مصابون بعدوي مرافقة بمرض نقص المناعة. إن كلا من الفحص المجهري وتحليل **Xpert® MTB/RIF** أمر جوهري لتشخيص الإصابة بمرض السُل في البلاد. إذ يجب أن تنصب جهود الفحص الطبي التشخيصية على جموع الذكور النشطين جنسيًا والمصابين بمرض نقص المناعة HIV والذين قد يشكلون مصدرًا لمرض سُل مقاوم أكثر للريفامبيسين من الإناث وذلك منعًا لانتشاره.

Translated from English version into Arabic by Norhan Mahmo, through

TRANSLATORS
WITHOUT BORDERS

评估 Xpert® MTB/RIF 试剂盒和镜检在纳米比亚结核杆菌诊断中的作用

Rooyen T. Mavenyengwa, Emma Shaduka, Innocent Maposa

摘要

引言：结核大约平均每年使 200 万人丧命并在世界范围导致 900 万人感染。对于该病正确的治疗，特别是资源有限的地区，往往受限于缺乏快速且容易操作使用的诊断工具。痰涂片镜检仍然是最廉价，最容易上手的诊断方法，但是该办法确诊的病例未占到艾滋病和结核病合并感染病例总数的一半，原因是细菌会扩散到肺以外的其他身体部位。全自动的 Xpert® MTB/RIF 试剂盒是非常具有前景的一个诊断工具，能同时检测利福平抗性。本研究旨在评估 Xpert® MTB/RIF 试剂盒和镜检在纳米比亚结核杆菌诊断中的作用，并以此来确定结核病的流行病学，计算感染结核病的比例，以及在整个国家中的疑似病例对利福平的抗性情况。

方法：本项回顾性研究分析了经 Xpert® MTB/RIF 试剂盒和镜检诊断的结核病例。数据来源于 2012 年 7 月到 2013 年 4 月纳米比亚病理学研究所的实验室信息系统登记的 13 个区的所有病例资料。

结果：本研究从具有肺结核的症状病例中共收集到 1 842 份标本。其中，594 (32.20%)份 Xpert® MTB/RIF 试剂盒检测为阳性，这部分中 443 例 (24.05%)镜检阳性，其余为阴性。与女性病例相比，男性病例对利福平的抗药性更高。

结论：肺结核在纳米比亚广泛分布，男性病例略多。大多数病例合并感染艾滋病。在纳米比亚，Xpert® MTB/RIF 试剂盒和镜检均具有重要作用。筛查诊断应该将重点放在对性活跃阳性 HIV 的男性群体——这部分人相对女性来说更加可能成为利福平抗药性的源头。

Translated from English version into Chinese by Xin-Yu Feng, edited by Pin Yang

Evaluation de l'analyse Xpert® MTB/RIF et de la microscopie pour le diagnostic de *la tuberculose Mycobacterium* en Namibie

Rooyen T. Mavenyengwa, Emma Shaduka, Innocent Maposa

Résumé

Contexte : La tuberculose (TB) tue environ deux millions de personnes et infecte environ neuf millions de personnes dans le monde chaque année. Sa bonne gestion, en particulier dans les pays à ressources limitées, a été entravée par le manque de tests de diagnostic rapides et faciles à utiliser. La microcopie frottis de cracha reste la méthode de diagnostic la moins chère et la plus facilement disponible, mais elle identifie uniquement la moitié des patients atteints de coïnfection VIH/TB parce que les bacilles auraient été diffusées dans les poumons vers d'autres régions du corps. L'analyse entièrement automatisée Xpert® MTB/RIF est une innovation prometteuse pour le diagnostic de la tuberculose et la détection de la résistance à la rifampicine. Cette étude vise à évaluer l'utilisation de l'analyse Xpert® MTB/RIF et de la microscopie pour le diagnostic de la *tuberculose Mycobacterium* en Namibie, en déterminant l'épidémiologie de la maladie et en calculant la proportion des cas infectés uniquement de la tuberculose et ceux qui ont une résistance à la rifampicine parmi le total des cas de tuberculose dans le pays.

Méthodes : cette étude rétrospective a analysé les cas de tuberculose qui ont été diagnostiqués en utilisant à la fois l'analyse Xpert® MTB/RIF et la microscopie. Les données ont été recueillies à partir des dossiers des patients à partir du système d'information de laboratoire Meditech de l'institut de pathologie de la Namibie pour la période de juillet 2012 à avril 2013. Les données de 13 régions ont été collectées.

Résultats : Le nombre total d'échantillons prélevés sur des patients présentant des symptômes de tuberculose pulmonaire était de 1 842. Parmi ceux-ci, 594 (32.20%) étaient positifs en utilisant l'analyse Xpert® MTB/RIF, dont 443 (24.05%) étaient également positifs en utilisant la microscopie. Le reste avait un résultat négatif. Les patients de sexe masculin étaient plus résistants à la rifampicine par rapport aux patients de sexe féminin.

Conclusions : La tuberculose est largement diffuse dans toute la Namibie, avec un peu plus d'hommes infectés que les femmes. La plupart des patients atteints de tuberculose sont également coinfectés par le VIB. La microscopie ainsi que l'analyse Xpert® MTB/RIF sont toutes deux cruciales pour le diagnostic de la tuberculose dans le pays. Les efforts de dépistage devraient se concentrer sur la population masculine séropositive sexuellement active qui pourrait être la source de plus de résistance au RIF TB que les femmes, pour prévenir sa propagation.

Translated from English version into French by ishaklamia, through

Оценка теста Xpert® MTB/RIF и микроскопии для диагностики микобактерий туберкулеза в Намибии

Руйен Т. Мавенйенгуа (Rooyen T. Mavenyengwa), Эмма Шадука (Emma Shaduka), Инносэнт Мапоза (Innocent Maposa)

Реферат статьи
Основные сведения: Ежегодно во всем мире из-за туберкулеза (ТБ) умирают около двух миллионов человек, а заражаются им около девяти миллионов. Надлежащее лечение туберкулеза затруднено в связи с отсутствием быстрых и простых в использовании диагностических тестов, особенно в условиях ограниченных ресурсов. Самым дешевым и легкодоступным методом диагностики остается микроскопия мазка мокроты, однако он определяет менее половины пациентов с коинфекцией ВИЧ/ТБ, так как бациллы распространяются из легких в другие органы. Полностью автоматизированный тест Xpert® MTB/RIF является многообещающей инновацией в диагностике ТБ и выявлении устойчивости к рифампицину. Целью этого исследования является оценка применения теста Xpert® MTB/RIF и микроскопии в диагностике микобактерий ТБ путем определения эпидемиологии заболевания в Намибии, а также подсчета доли случаев инфицирования только ТБ и случаев устойчивости к рифампицину среди общего числа случаев подозреваемого ТБ в стране.
Методы: Это ретроспективное исследование проанализировало случаи заболевания ТБ, диагностированного как с помощью теста Xpert® MTB/RIF, так и с помощью микроскопии. Данные были собраны из историй болезней, хранящихся в лабораторной информационной системе "Медитек" Института патологии Намибии и относящихся к периоду с июля 2012 г. по апрель 2013 г. Эта информация касалась 13 регионов.
Результаты: Всего взято 1842 пробы у пациентов с симптомами ТБ легких. Тест Xpert® MTB/RIF обнаружил MTB в 594 пробах (32,2%). В 443 пробах (24,05%) МТБ были обнаружены с помощью микроскопии. В остальных пробах МТБ не обнаружены. Пациенты мужского пола были более устойчивы к рифампицину по сравнению с пациентами женского пола.
Заключение: ТБ широко распространен на всей территории Намибии, при этом число зараженных мужчин превосходит число зараженных женщин. Большинство больных ТБ также инфицированы ВИЧ. Как микроскопия, так и тест Xpert® MTB/RIF исключительно важны для диагностирования ТБ в стране. Для предотвращения распространения ТБ, устойчивого к рифампицину, основное внимание следует уделять скрининговому обследованию сексуально активных ВИЧ-позитивных мужчин, которые более, чем женщины могут быть источником устойчивого к рифампицину ТБ.

Translated from English version into Russian by Larysa K., through

TRANSLATORS
WITHOUT BORDERS

Evaluación de la prueba Xpert® MTB/RIF y microscopía para el diagnóstico de la *Mycobacterium tuberculosis* en Namibia

Autores: Rooyen T. Mavenyengwa, Emma Shaduka, Innocent Maposa

Resumen
Contexto: Aproximadamente dos millones de personas mueren de tuberculosis al año en todo el mundo, mientras que unos nueve millones contraen la enfermedad durante dicho periodo. La gestión adecuada de la tuberculosis, especialmente en lugares con recursos son limitados, se ha visto dificultada por la falta de pruebas diagnósticas fáciles y rápidas de realizar. La microscopía de esputos sigue siendo el método de diagnóstico más barato y accesible, pero solo logra identificar a menos de la mitad de los pacientes con VIH, ya que el bacilo se extiende de los pulmones a otras partes del cuerpo. La prueba Xpert® MTB/RIF, totalmente automatizada, se revela como una innovación prometedora para diagnosticar la tuberculosis y detectar la resistencia a la rifampicina. El objetivo de este estudio es evaluar el uso de la prueba Xpert® MTB/RIF y la microscopía en el

diagnóstico de la *mycobacterium tuberculosis* en Namibia, determinando la epidemiología de la enfermedad y calculando la proporción de casos de infección solo por tuberculosis y aquellos con resistencia a la rifampicina de entre todos los presuntos casos de tuberculosis del país.
Metodología: Este estudio retrospectivo analizó los casos de tuberculosis diagnosticados tanto mediante microscopía como con la prueba Xpert® MTB/RIF. Los datos se obtuvieron de los historiales médicos del sistema de información del laboratorio Meditech del Instituto de Patología de Namibia, durante el periodo comprendido entre julio de 2012 y abril de 2013. Se recopilaron datos de trece regiones del país.
Resultados: El total de muestras recogidas de pacientes con síntomas de tuberculosis pulmonar fue 1.842. De estos, 594 (el 32,2 %) dieron positivo en *mycobacterium tuberculosis* en la prueba Xpert® MTB/RIF. De ese grupo, 443 (el 24,05 %) también dieron positivo en la microscopía, mientras que el resto dio negativo. Los hombres presentaron mayor resistencia a la rifampicina en comparación con las mujeres.
Conclusiones: La tuberculosis se distribuye por toda Namibia, con una cifra de infectados ligeramente mayor que de infectadas. La mayoría de los pacientes también estaban infectados de VIH. En el país, tanto la microscopía como la prueba Xpert® MTB/RIF son cruciales para diagnosticar los casos de tuberculosis. A la hora de prevenir el contagio, los esfuerzos de diagnóstico y monitorización deberían centrarse en la población masculina con VIH sexualmente activa más que la femenina, ya que el anterior grupo demográfico podría engendrar una tuberculosis más resistente a la rifampicina.

Translated from English version into Spanish by Marta Callava Linares, through

TRANSLATORS
WITHOUT BORDERS
